# Maximal viral information recovery from sequence data using VirMAP

**DOI:** 10.1038/s41467-018-05658-8

**Published:** 2018-08-10

**Authors:** Nadim J Ajami, Matthew C. Wong, Matthew C. Ross, Richard E. Lloyd, Joseph F. Petrosino

**Affiliations:** 1 Alkek Center for Metagenomics and Microbiome Research, Baylor College of Medicine, Houston, TX 77030 USA; 2 Department of Molecular Virology and Microbiology, Baylor College of Medicine, Houston, TX 77030 USA

## Abstract

Accurate classification of the human virome is critical to a full understanding of the role viruses play in health and disease. This implies the need for sensitive, specific, and practical pipelines that return precise outputs while still enabling case-specific post hoc analysis. Viral taxonomic characterization from metagenomic data suffers from high background noise and signal crosstalk that confounds current methods. Here we develop VirMAP that overcomes these limitations using techniques that merge nucleotide and protein information to taxonomically classify viral reconstructions independent of genome coverage or read overlap. We validate VirMAP using published data sets and viral mock communities containing RNA and DNA viruses and bacteriophages. VirMAP offers opportunities to enhance metagenomic studies seeking to define virome-host interactions, improve biosurveillance capabilities, and strengthen molecular epidemiology reporting.

## Abstract (web summary)

Viral taxonomic characterization from metagenomic data suffers from high background noise and signal crosstalk. Here, the authors develop VirMAP, a novel pipeline for analyses of metagenomic data that classifies viral reconstructions independent of genome coverage or read overlap.

## Introduction

Accurate classification of viral sequences in metagenomic datasets remains challenging despite large advances in next-generation sequencing and bioinformatics. Current methods rely on the classification of individual reads and/or contigs through either alignments^1–8^ or kmer analyses^9,10^. These approaches are all restricted by similar problems including: reads, contigs, or kmers mapping equally well to different genomes, codon degeneracy allowing two low-identity nucleotide sequences to code identical polypeptides, widely varying sequence coverage impacting assembler heuristics, large amounts of contaminating sequences, and databases lacking sufficient coverage of the viral tree of life. While some of these issues are solved by generating high-quality contigs from an assembly, achieving the necessary coverage for a quality assembly remains challenging. These issues are particularly relevant to clinical and environmental virology as they prevent accurate characterization of viruses from sequence data alone.

**Fig. 1:**
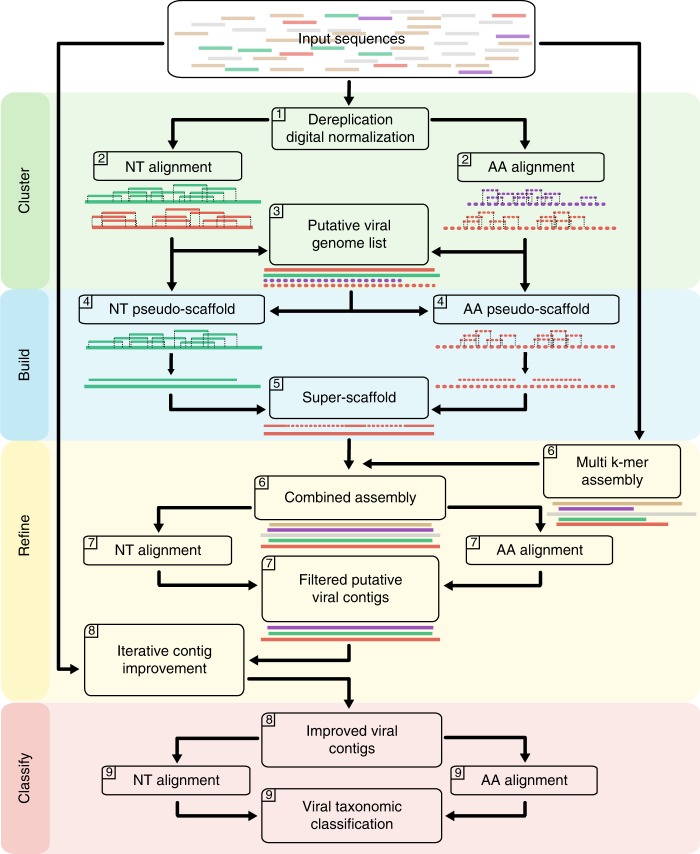
A schematic overview of VirMAP. Data processing with VirMAP is achieved through four main stages (shaded colors) divided into nine major steps (top left corner). A putative list of viral genomes and protein pseudo-scaffolds are constructed from *clustered* nucleotide and translated alignments to the Genbank viral and phage divisions (gbvrl and gbphage). Nucleotide and amino acid pseudo-scaffolds are “built” and merged into a single super-scaffold per genome. A merged de novo assembly is constructed and merged in, resulting in contigs that are then *refined* using an iterative rebuild process. The improved dual assembly is filtered against a comprehensive Genbank database and are taxonomically *classified* using a novel per-base contig scoring system

We here develop VirMAP to address the current challenges in identification and classification of low coverage and highly divergent viruses within metagenomic datasets. VirMAP uses a variety of techniques including a mapping assembly algorithm combining nucleotide and amino-acid alignment information in a tiered manner to build virus-like super-scaffolds, a merging system designed to hybridize a mapping assembly and a de-novo assembly, an improvement algorithm that iteratively merges and rebuilds contig information, and a taxonomic classification algorithm that is centered around a bits-per-base scoring system (Fig. 1).

## Results

### Test data sets

To evaluate the performance of VirMAP, we chose four publicly available datasets that encompass common scenarios faced when determining viral taxonomies (e.g., high vs low coverage, high vs. low database homology, high vs. low non-viral background). We also performed the same analysis using a more standard approach described in the online methods to compare VirMAP’s output, runtime, and computational results (Supplementary Data 1 and Supplementary Note 1). Finally, to evaluate the impact of genome coverage in the classification of viral taxonomies, we ran all samples subsampled at 10%, 1%, and 0.1%, 10 times each. For the results discussed below, VirMAP did not report any taxonomies with <1000 bits of aggregate alignment information across all contigs, unless otherwise noted.

### Mock Communities

We generated a single viral mock community (VMC) (BioProject ID PRJNA431646) by combining purified preparations of seven different viruses (human mastadenovirus B, human mastadenovirus C, murine gammaherpesvirus 4, coxsackievirus B4 [strain Tuscany], echovirus E13 [strain Del Carmen], human poliovirus type 1 [strain Mahoney], and rotavirus A) at different concentrations in phosphate-buffered saline. VMC was extracted, reverse transcribed with random hexamers, amplified using barcoded semi-random primers, and sequenced on the Illumina HiSeq2500 platform (2 × 150 bp) at varying sequencing depths yielding 36,974,536 total reads (Methods). Following standard adapter trimming and filtering (hg38 and PhiX), 6,180,026 total reads remained. The majority of reads were filtered using BBDuk due to a high presence of adapter dimers and human genome contamination (41.75%) likely a consequence of low DNA concentration in the library preparation.

**Fig. 2:**
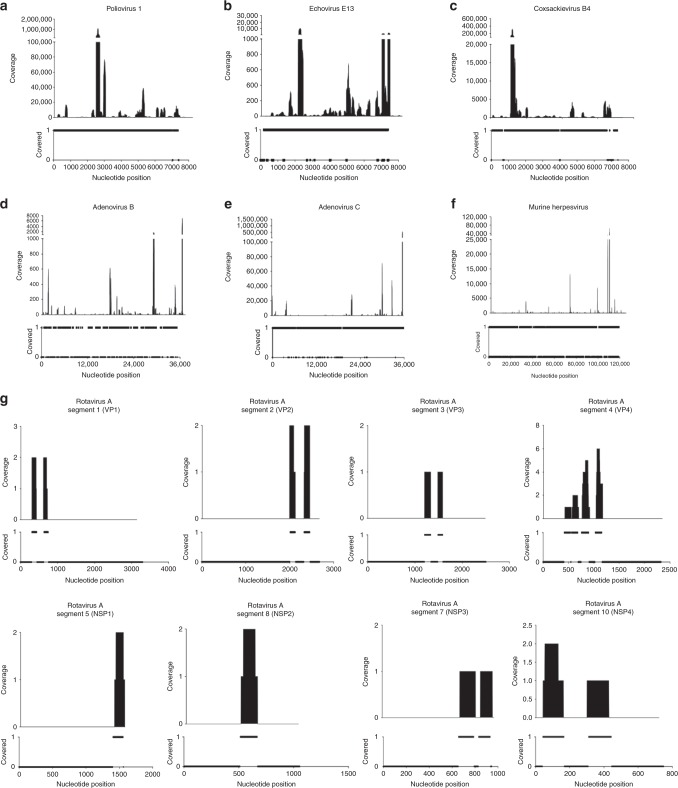
Viral Mock Community (VMC) calculated genome coverage depth and span from remapping source reads to VirMAP reconstructed genomes. The VMC consists of purified preparations of seven different viruses (**a**) human poliovirus type 1 [strain Mahoney], (**b**) echovirus E13 [strain Del Carmen], (**c**) coxsackievirus B4 [strain Tuscany], (**d**) human adenovirus (**b**, **e**) human adenovirus (**c**, **f**) murine gammaherpesvirus 4, and (**g**) rotavirus, combined at different concentrations in phosphate-buffered saline. Coverage depth and span are represented for each of the viruses in VMC per nucleotide position. For coverage span, a value of 1 represents a nucleotide position covered with respect to the source genome. VMC is available at BioProject ID PRJNA431646

VirMAP assigned 50.1% (3,099,015) of quality trimmed, adapter trimmed, and human-filtered VMC reads to viruses. Of these, 99.99% were assigned to the following: human poliovirus 1 (47.04%), human mastadenovirus C (30.90%), coxsackievirus B4 (13.30%), murid gammaherpesvirus 4 (7.43%), echovirus E13 (0.78%), human mastadenovirus B (0.55%), and rotavirus A (32 reads, 0.0010%) (Fig. 2 and Supplementary Data 2). The remaining 8 reads were assigned to bosavirus MS-2016a, which is thought to be a cell culture contaminant present in fetal bovine serum. The low abundance of rotavirus reads corresponded to the low titer used to build the VMC. A random subsample and mapping of the remaining reads not classified by VirMAP showed high homology to cell culture and mouse host cell lines. No further investigative efforts were performed.

At 10 and 1% subsampling, VirMAP reported six of seven viral constituents in all trials, with rotavirus remaining unreported (Supplementary Data 2). At 0.1% subsampling, VirMAP reported six of seven viral constituents in eight of the ten trials, rotavirus remained unreported in all trials. The two trials reporting only five members missed adenovirus B. In all subsampled trials at all depths, no viruses other than the expected members of the VMC were reported by VirMAP.

In addition to processing the VMC dataset with VirMAP, we applied a standard taxonomic classification method (see “methods” section) for comparative purposes. The standard approach reported eight taxonomies. It missed rotavirus but included an unidentified adenovirus and a misclassified adenovirus taxonomic call, likely misclassifications of human mastadenovirus B contigs (Table 1 and Supplementary Data 1). All subsampling trials using the standard approach failed to report rotavirus, similar to VirMAP. At 10% subsampling, the standard approach reported six of seven members of the VMC in only two of ten trials. At 1% and 0.1% subsampling, the standard approach reported no more than three and two members of the VMC, respectively (Supplementary Data 3).

**Table 1:** Comparative analysis of ten viral sequence classifiers

Pipeline	Mapped Reads (%)	Unique Calls	Viral Taxonomies	CCR (% of mapped)	Precision	Recall	F-score
VirMAP	3,099,015 (50.1%)	8	8	3,099,007 (99.999%)	0.88	1.00	0.94
Read classification							
FastViromeExplorer	2,710,170 (43.85%)	7	4	2,710,170 (100%)	1.00	0.57	0.73
VirusSeeker^a^	10,750 (0.174%)	16	16	1,467 (13.65%)	0.31	0.57	0.40
Kaiju	2,287,962 (37.02%)	227	227	433,243 (18.94%)	0.09	1.00	0.17
ViromeScan	663,185 (10.73)	427	354	614,016 (92.586%)	0.01	0.57	0.02
Contig classification							
drVM^b^	22,404,813 (362.54%)	673	158	18,235,876 (81.39%)	0.35	1.00	0.52
VirusTAP	NA	5	5	NA	0.6	0.43	0.50
VIPIE^c^	~109633 (~1.77%)	13	11	~23,731 (~21.65%)	0.30	0.71	0.42
Standard method^d^	2,319,573 (37.53%)	8	8	2,273,193 (98.03%)	0.75	0.86	0.80
Marker gene classification							
MetaPhlAn2	NA	5	5	NA	0.40	0.29	0.34

The Viral Mock Community (VMC) dataset (6,180,026 trimmed reads) was processed through nine different pipelines for viral taxonomic classification. VMC was generated by combining purified preparations of seven different viruses (human adenovirus B, human adenovirus C, murine gammaherpesvirus 4, coxsackievirus B4 [strain Tuscany], echovirus E13 [strain Del Carmen], human poliovirus type 1 [strain Mahoney], and rotavirus A) in phosphate-buffered saline. Unique calls refer to the distinct database entries reported while viral taxonomies represent a reduction of unique calls to NCBI taxonomic ID. CCR: Correctly Classified Reads. Precision: (true positives/true positives + false positives). Recall: (true positives/true positives + false negatives), F-score: harmonic average of recall and precision scores 2 × ((*P* × *R*) / (*P* + *R*))

^a^VirusSeeker applies filtering and clustering techniques to the reads and final counts are derived from this reduced set

^b^drVM internally counts identical reads across multiple reported entries, so the total counts can exceed 100%

^c^VIPIE reports reads as counts per 100,000 reads, the approximation is a rescaled amount against the original read counts

^d^The standard approach employs a metagenomic assembly using MEGAHIT and a sequential top-hit mapping classification using BLASTn and BLASTx

The total number of viral bases reconstructed by VirMAP (162,705 bp) relative to the full VMC contig set decreased to 55.55, 25.99, and 10.0% for 10, 1, and 0.1% subsampling levels, respectively. The total viral bases constructed and identified by the standard approach (60,073 bp) decreased to 24.76, 5.36, 0.11% for 10, 1, and 0.1% subsampling levels, respectively. These results highlight VirMAP’s ability to more correctly build viral genomic reconstructions and assign viral taxonomies despite declining genomic coverage. In addition to VirMAP and the standard assembly and mapping approach, we also employed publicly available taxonomic classifiers that rely on read mapping (FastViromeExplorer^5^, VirusSeeker^8^, Kaiju^9^, and ViromeScan^3^), contig mapping (VirusTAP^7^, VIPIE^2^), a combination of both read and contig mapping (drVM^1^), or marker gene mapping (MetaPhlAn2^6^), for comparative purposes using the VMC dataset (Table 1). Other existing pipelines (e.g. metaVIC^11^, VIROME^12^, VIP^13^, and MetaVir2^14^) were not included in our analysis due to unavailability of essential resources such as missing databases and discontinued web resources.

Using the list of intended viral constituents in VMC, we evaluated the number of unique viruses and total taxonomic IDs found to calculate values for precision, recall, and F-score for each pipeline. Simultaneously, the total number of reads used and the total number of correctly classified reads were tracked. Precision, recall, and F-score values for VirMAP were 0.88, 1.0, 0.94, respectively, on VMC. The recall value indicated that VirMAP was able to find all expected constituents of the mock community, however, the presence of a single viral contaminant lowered the precision value. The F-score shows that VirMAP was highly accurate. CPU hours used were not tracked as not all methods are were locally operated. Contig classifying approaches generally had higher precision values as aligning longer queries minimizes the chance of a colliding alignment with database entries containing short errors, e.g., flanking vector sequences. However, such approaches are not likely to find low abundance viruses due to most assembler’s inability to make contigs from non-overlapping reads. Read classifying approaches generally suffer more from aberrant database alignments and must implement effective filters to overcome these errors to achieve higher precision scores. FastViromeExplorer’s use of the ratio between observed and expected coverage effectively filters a large amount of noise, resulting in an excellent precision value. However, read count and taxonomic rank cutoffs lowered its recall value, similar to most of the other read classifying pipelines. Marker gene mapping methods offer a separate solution to classifying taxonomies while overcoming database contamination issues; however, they are heavily reliant on properly annotated genome entries as well as high coverage. In order to effectively find viruses, we believe a combination of both read and contig mapping approaches must be effectively used.

**Fig. 3:**
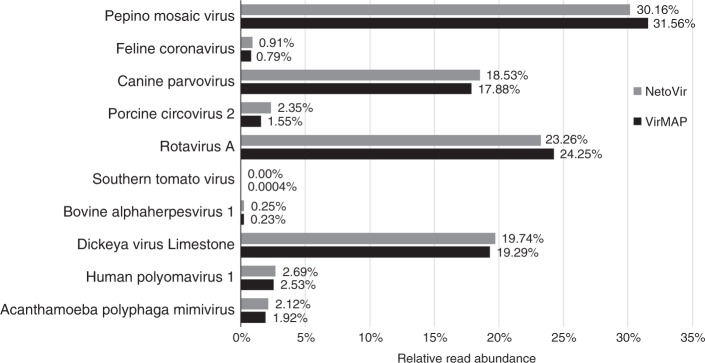
VirMAP analysis of an external mock community. A mock virome control sample (SRR3458562) recently reported^15^ was processed with VirMAP. A total of 5,969,272 reads (32.96%) were classified as being of viral origin across 10 distinct viral lineages which included the nine viral constituents of the mock community. Additionally, one putative contaminant virus was identified: southern tomato virus

To validate the VMC result, a wholly separate mock virome was evaluated. The novel enrichment technique of viromes (NetoVir) method for sample preparation was recently described employing a comprehensive mock viral community of nine viruses^15^, and the resulting dataset was made publicly available (BioProject: PRJNA319556). This mock virome consists of known concentrations (ranging from 10^7^ to 10^10^ genome copies per mL) of nine viruses spanning diverse taxonomic, genomic, and physical characteristics. We selected the control sample (SRR3458562), as it represented the least processed dataset, and analyzed it with VirMAP. A total of 6,707,800 reads (37.04%) were classified as being of viral origin across 10 distinct viral lineages, which included the nine viruses in the mock community (Fig. 3). One additional virus identified in the set was southern tomato virus, a virus previously observed to co-infect with pepino mosaic virus^16^, a member of the mock community. Overall, a good correlation was observed between the proportion of viral reads per lineage obtained through VirMAP and those reported by Conceição-Neto (*R*^2^ = 0.9972)^15^.

Following a similar approach to the subsampling strategy with VMC, the NetoVir dataset was subsampled at 10, 1, and 0.1%, 10 times each, and analyzed using VirMAP and the standard approach. In all 10 trials at each subsampling depth, all nine mock community viruses were correctly reported by VirMAP except for a single trial at 0.1% subsampling where bovine alphaherpesvirus 1 was missed (Supplementary Data 4). Viruses other than those present in the mock community were reported by VirMAP. In five trials at 10% and five trials at 1% subsampling, a contig likely belonging to Dickeya virus Limestone was not incorporated into the main Dickeya virus contig and was subsequently underclassified as “myoviridae”. The unincorporated contig maps across the 5′ and 3′ ends of the pseudo-constructed genome, suggesting a circular genome or chimeric misassembly. However, VirMAP’s merging engine assumes a linear genome and is unable to incorporate contigs in this fashion. In one trial at 10%, 16 reads likely belonging to acanthamoeba polyphaga mimivirus were classified as “mimivirus”. In one trial at 10%, 10 reads from southern tomato virus were reported. Finally, in one trial at 1%, 26 reads likely belonging to Dickeya virus Limestone were reported as “unclassified phage”. This error was traced to two database entries, sharing 99% sequence identity to Dickeya virus Limestone, that were misannotated (see Supplementary Note 2 for further discussion).

For the standard approach at 10% subsampling, eight trials reported all nine members of the community, with one trial missing feline panleukopenia virus and one trial missing human polyomavirus 1. At 1% subsampling, all ten trials missed mimivirus, and eight of ten trials missed bovine alphaherpesvirus. At 0.1%, no trial reported more than six of the nine expected viruses. The standard approach reported an average of 5.5, 2.8, and 1.1 erroneous taxonomic IDs per trial at 10, 1, and 0.1% subsampling, respectively (Supplementary Data 5).

### Published metagenomic data sets

To test VirMAP using clinical samples, we processed a dataset (submitted by University Medical Center Hamburg-Eppendorf on 2015-05-22 under the title Unbiased metagenomic RNA sequencing (UMERS)) of 20 respiratory specimens (BAL, sputum and swab) of patients with seasonal influenza infection and five BAL samples from patients with influenza-negative pneumonia, (BioProject ID PRJEB7888)^17^.

The dataset contained 25 samples totaling 178 Gb represented by 1.75 billion reads generated with both Illumina HiSeq (2 × 100 bp) and MiSeq (2 × 150, 2 × 250, and 2 × 300) chemistries. Of these, 2,430,488 (0.14%) were determined to be viral and 327,940 reads were incorporated into contigs greater than 500 bp that were unclassifiable by VirMAP (Supplementary Data 6). All individual fastq sets were concatenated based on biosample id and results were compared to those reported by the authors who used qPCR and sequencing to evaluate the samples.

VirMAP was able to reconstruct contigs representing not only all viral genome segments reported by the authors, but also 32 influenza contigs across four samples for which the authors did not report findings (sample IDs 677, 768, 1116, and 1689) (Supplementary Data 7). Across all publicly available samples, VirMAP recruited 193% more reads to contigs determined to be influenza compared to the authors (UMERS - 263,210 reads, VirMAP - 509,039 reads).

In two instances, the strongest viral signal determined by VirMAP disagreed with the reported one (samples 677 and 768). In both cases, positive H1N1 qPCR results were reported. These two samples represented the two highest CT values observed for the H1N1 specific probe (both CT of 34). In sample 677, VirMAP detected nine influenza A segments and assigned seven segments to H3N2, one segment (genome segment 4) to H1N1, and one to Influenza A. This likely explains the authors positive H1N1 result as the qPCR probe targets genome segment 4. In sample 768, VirMAP partially reconstructed eight influenza A genome segments, assigning all of them to H3N2. However, the signal in this sample was low, consisting of only 60 total reads. The positive H1N1 qPCR indicates that an H1N1 genome may have been present at a level below the limit of detection for sequencing.

Additionally, VirMAP determined sample 104 to have the strongest H1N1 signal among all samples tested based on the number of recruited reads. However, the authors reported a negative H1N1 qPCR result for this sample. Mapping the probe sequence against VirMAP’s reconstructed H1N1 segment 4 shows a mismatch at base 15 of the probe sequence, potentially explaining their negative result.

The authors also report five samples containing viruses other than influenza. VirMAP found the same viruses in three out of five samples. The data for the remaining two reported samples were not among the publicly available files. In addition to non-influenza viruses reported by the authors, VirMAP was able to detect human herpesvirus 4 in two samples, human parainfluenza virus 3 in a sample other than the one reported by the authors, and astrovirus HK2014 in one sample.

The standard approach for this dataset yielded an average of 6.13 influenza segments per sample across 16 of the 20 known positive samples, with the remaining four samples producing no results, mirroring the authors original result. Segments produced by the standard approach consisted of 1.07 contigs per segment on average (Supplementary Data 8). For comparison, VirMAP yielded an average of 9.05 influenza segments per sample across all 20 samples. All segments reconstructed by VirMAP consisted of one contig per segment. The average number of segments reported by VirMAP exceeded the number of Influenza genome segments because VirMAP identified multiple influenza virus subtypes per sample across the majority of samples. The extra segments were found in trace amounts and below the threshold that any assembler could use to generate a contig.

**Fig. 4:**
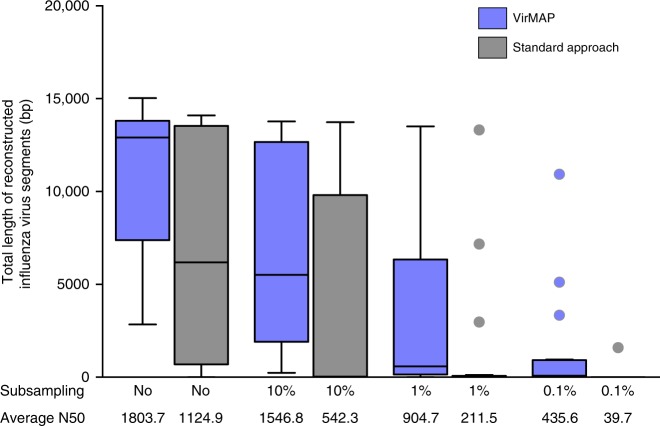
A comparison of VirMAP and the Standard Approach using the influenza virus dataset (BioProject ID PRJEB7888). The total length of reconstructed influenza virus segments was calculated at different levels of subsampling by adding the total number of base pairs found for across segments. An average N50 was calculated at each subsampling level by averaging the N50 values for all trials (20 at 100%, 200 at 10, 1, and 0.1). The percentage of positive trials correspond to the ratio of trials with >1 identifiable influenza contig over the total number of trials (20 at 100%, 200 at 10, 1, and 0.1%). Tukey plots, bar: statistical median, edges: low, 25%; high 75% quartiles, whiskers:1.5 × interquartile range, dots: outliers)

The Influenza dataset was subsampled using the same conditions as the mock communities described above. At 10% subsampling, the standard approach reported influenza in 10 out of 20 samples in at least one trial. For trials where influenza was found, an average of 5.23 influenza segments were reported. The remaining 10 samples failed to produce influenza contigs in any trial. Using the same dataset, VirMAP reported influenza across all 20 samples in at least one trial. For trials where influenza was found, an average of 6.55 influenza genome segments were reported. At 1% subsampling, the standard approach, when finding influenza, averaged 2.73 segments across 7 samples, while VirMAP, when finding influenza, averaged 4.22 segments across 18 samples. At 0.1% subsampling, the standard approach, when finding influenza, averaged 2.1 segments in one sample, while VirMAP, when finding influenza, averaged 2.23 segments across 17 samples. However, in two samples the influenza signal was below the default aggregate cutoff of 1000 bits per taxon, and thus would not have been reported. (Supplementary Data 9). We compared the total reconstructed sequence length of influenza segments between VirMAP and the standard approach at all levels of subsampling. Results show VirMAP improves both the length and contiguity of influenza segments when using the total dataset and at all levels of subsampling (Fig. 4).

These results highlight drawbacks of assembly-based classification methods, primarily the dependency on overlapping read segments, which precludes assembly of low abundance viruses. As VirMAP combines information derived from both read mapping and read assembling, it is able to extract and classify viral information even if the underlying coverage of a virus is well below the abilities of a de-novo assembly engine. This is especially apparent in the 0.1% subsampling set; VirMAP still reported the influenza virus in 17 out of 20 samples, while the standard approach reported influenza virus in only one.

To test VirMAP against a more complex metagenomic dataset, we used a data set submitted by the University of Brasilia on 01 August 2017 under the project title “Viral diversity of the Federal District of Brazil” (BioProject ID PRJNA395784). This dataset was chosen due to its recent submission and potentially complex viral community composition. Four samples totaling 19.23 Gb represented by 199.67 million reads generated with Illumina (2 × 100 bp) chemistry from a TruSeq RNA library with a ribosomal depletion step were processed through VirMAP. Of these, 4,932,775 (2.47%) were determined to be viral and 2,062,096 million reads were incorporated into contigs greater than 500 bp that were unclassifiable. A total of 489 viral taxonomies were identified in all four samples, of which Laverivirus UC1 captured the most reads (1,046,272 reads). The virus reported in all four samples that captured the most reads (428,511) was maize rayado fino virus. Sample SRR5868318 contained a high prevalence of human enteric viruses and was collected in June of 2014 (Supplementary Data 10).

This dataset was also processed with the standard assembly and mapping approach described previously. Initial standard results were filtered to remove taxa IDs with <300 aggregate bits of alignment, which lowered the average number of taxa IDs reported per sample from 480 to 236 (Supplementary Data 11 and Supplementary Data 12). Three-hundred bits of aggregate alignment information was empirically chosen as a filtering cutoff for the standard approach since the number of taxa containing only contigs with very weak (50–100) bit-scores increased substantially below that threshold. This suggests that the majority of taxa IDs found by the standard approach below that threshold are potentially very low information hits. For VirMAP, the internal pipeline filters all viral contigs below 300 bits of information, and the standard table construction script further filters taxa IDs that score below an aggregate 1000 bits of alignment information. 300 bits is the default threshold as contigs containing less aggregate alignment information were commonly observed to be false positives. VirMAP found an average of 423 taxa IDs per sample in the raw unfiltered output (300 bits/contig minimum), and reported an average of 231 taxa IDs per sample in the filtered table output (1000 bits/taxa ID minimum). As these samples are environmental, there is no underlying ground truth to compare against. The standard approach found 4.3 megabases of contigs that could be mapped to a viral taxonomy; when filtered at 300 aggregate bits per taxon, 4.0 megabases of contigs could be mapped to a viral taxonomy. These results suggest that the taxa IDs filtered by the 300 bit threshold are from short contigs and not longer contigs containing short and weak alignments to viral sequences. VirMAP in its raw output was only able to construct 1.69 megabases of contigs, and 1.04 megabases of contigs when filtered. The disparity between total contig length reported is likely caused by the standard assembly creating longer low coverage contigs that have slight homology to known viral proteins, as only the top scoring alignment per contig is used for classification. One example would be a prophage embedded in highly divergent bacteria serving as a contig’s best alignment event. Such a contig would contribute its full length to the total length of viral contigs on the weight of a single low query coverage alignment event. Contrasting with VirMAP’s strategy to use as much alignment information as possible when calling a contig’s taxonomic origin, this issue causes the standard approach to be prone to false positives, especially in low scoring situations. The amount of contigs per taxa ID in the filtered datasets was 2.29 for VirMAP and 12.25 for the standard approach. For the raw datasets, VirMAP assigned 1.81 contigs per taxa ID while the standard approach assigned 6.94. This shows that the standard approach created more contigs containing only low scoring alignments to a single taxa ID. Upon filtering, the remaining taxa IDs with higher amounts of alignment information are much more fragmented on average. VirMAP exhibits this behavior as well, albeit at a much lower rate.

The four samples from the Brazilian sewage set were subsampled according to the same conditions as the mock communities and Influenza dataset. When unfiltered, the standard approach yielded an average of 274 taxa per sample per trial at 10% subsampling (Supplementary Data 13). The 1 and 0.1% trials yielded an average of 68 and eight taxa, respectively. When filtered, the standard approach yielded an average of 112, 30, and 4 taxa per sample per trial for the 10, 1 and 0.1% trials, respectively. VirMAP averaged 453, 189, and 77 taxa per sample per trial for the unfiltered tables, and an average of 174, 62, and 24 taxa per sample per trial for the filtered tables of the 10, 1 and 0.1% trials, respectively. For the 10% unfiltered set, VirMAP reported more taxa IDs than the non-subsampled unfiltered run. An inspection showed this to be caused by different random portions of low coverage, low identity viruses not containing enough aggregate alignment information to enter the LCA engine, resulting in an overclassification of contigs. However, when filtering using the default 1000 bit threshold this artifact disappeared. Comparing either filtered or unfiltered tables, the drop-off in detectable taxa when subsampling was not as steep with VirMAP, suggesting a more gradual drop off in the retrieval of meaningful information at increasingly suboptimal coverage levels. A qualitative comparison on how the pipelines presented in Table 1 performed on the Influenza and Brazil datasets can be found in Supplementary Note 3.

## Discussion

Here we present a pipeline for viral metagenomics that aims to maximize viral information recovery and reconstruction to provide the highest level of taxonomic resolution possible. It operates in a manner independent of coverage depth and offers parameters that can be modified to tune the sensitivity/specificity tradeoff (Supplementary Data 14). VirMAP was created out of the necessity to accurately define viral taxonomies from clinical and environmental samples which lacked closely related database entries. This pipeline provides a unique methodology for viral detection and classification with relevance in clinical virology, viral surveillance, and molecular epidemiology. Overall, VirMAP offers a major advancement over existing viral classification pipelines in the accurate detection and identification of viral sequences from metagenomic datasets and provides the user with intermediate and final outputs to continue post hoc investigation for case-specific studies.

## Methods

### Contig generation

VirMAP employs both de-novo and mapping assembly strategies to create a working set of contigs. For the mapping arm, input reads are dereplicated and digitally normalized^18^ using default parameters. These two steps reduce the amount of data used while minimizing information loss; however, they are not required. Processed reads are aligned to a comprehensive viral nucleotide and viral protein database sourced from the Genbank viral (gbvrl) and phage (gbphg) divisions using BBMap^19^ and DIAMOND^20^, respectively. All translated alignments within 20% of the subject sequence and within 8% of the top scoring subject per query are reported. For nucleotide alignments the thresholds are 90% and 5%, respectively. These thresholds were empirically found to minimize false positive centroids in the downstream clustering step. Reads are assigned to viral genomes by nucleotide and translated alignments resulting in a set of reads per genome. Genomic read sets are then filtered by Jaccard distance if high numbers of non-unique read membership are observed relative to larger sets. Each nucleotide and amino acid entry associated with a non-filtered genome is individually reconstructed using a naive pileup assembly. Reconstructed protein coding sequences are tiled on top of their originating genome via their annotated CDS positions. Disagreements within an assembly are masked. If there are disagreements between the nucleotide and amino-acid mapping assemblies, precedence is given to the nucleotide call. The entire process creates a pseudo-assembled nucleotide super-scaffold representing coherently assembled viral information. VirMAP’s mapping reconstruction performs well even in situations where an underlying genome has both low read depth and high divergence from the nearest known databaszae entry. For the de-novo arm, the full read set undergoes a multi-kmer de-novo assembly using either MEGAHIT^21,22^ or Tadpole^19^, or both. This step can provide secondary confirmation of the pseudo-constructed contigs and can allow viral genomes without any nearby database entry to potentially be constructed if coverage is sufficient.

### Iterative assembly improvement

All constructed contigs are mapped to a custom database described in the Taxonomic determination section. Contigs that map outside the viral superkingdom are filtered. Filtering is accomplished with the same technique used in the taxonomic determination step described below. Dereplicated reads are mapped to the remaining contigs and a continuous cycle of merging, mapping, piling up, and re-assembling is carried out until all contigs converge. Merging is accomplished by iteratively mapping the contigs to themselves and joining any two with significant overlap. Reassembly is accomplished using a naive pileup mapping assembly of the dereplicated, non-normalized read set. After convergence, the reads are mapped to the final set of contigs and read abundance is calculated.

### Taxonomic determination

To determine the taxonomic origin of a contig, VirMAP employs a two-pass taxonomic scoring algorithm that considers alignments in terms of maximum bit-score per base achieved by a subject taxon for each contig, as well as the relative bit-score per base for each subject entry per taxon weighted against the best scoring taxon at each position. Pass one calculates a maximum achieved bit score per base for each subject taxon by dividing the bit score by the aligned length for each alignment event and applying the fractional score to the subject taxon across the bases covered. Top scores per taxon and globally across all taxa are tracked for every covered base position. The top scores for each subject taxon across all positions covered by the taxon’s alignment events are summed up and represent a taxon’s best potential alignment independent of subject contiguity. In pass two, once the global top scores per position are known, each subject alignment’s bit score per base is weighted against the global top score per position. All second pass weighted scores per subject entry are summed up for each taxon across all base positions. This second pass creates a secondary store of information akin to an aggregate overall identity of a taxon’s database entries relative to the contig in question. Essentially, the scoring algorithm considers the best possible alignment of a query against an individual taxon (pass one), as well as the aggregate relative accuracy of all alignments achieved by a taxon (pass two) as two independent scores per query. This helps in events where a small amount of misclassified entries in a taxon are slightly closer to the contig in question relative to the true taxon of origin. By using the secondary database “volume,” misclassified entries have a much lower chance of causing a contig classification error as all of the neighboring entries in their taxon will contribute much lower amounts of “volume.” All scores are independently calculated from nucleotide and translated alignments of the improved contigs to a comprehensive Genbank database spanning 16 separate divisions (Supplementary Note 4). Pass two nucleotide scores are weighed down exponentially while protein scores are weighed down tetrationally. Since protein sequences are generally more conserved, any mismatch in amino acid alignment is penalized more heavily. A contig is filtered if its top scoring pass one taxon is not in the superkingdom virus, or if > 50% of its aligning bases do not resolve to the superkingdom virus. All pass one taxa scoring within a dynamically calculated radius per contig are considered in a rank-wise lowest common ancestor (LCA) algorithm. The radius varies based on the best scoring taxon relative to a hypothetical maximum scoring self-alignment as well as the total bits of information possible given the maximum self-score. In the LCA engine, each taxon contributes its pass two score to its parent taxon if there is no taxon scoring above a dynamically calculated majority threshold at the current rank. The threshold is calculated by the total amount of database “volume” calculated in pass two across all taxa entering the LCA. As the total input database volume increases, the amount of database volume required above a 50% majority decreases. Thus, if a viral taxon is poorly represented in the database, a higher threshold is required to assign taxonomy. Conversely, if a taxon is very well represented, only a slight majority is required. If no taxon exceeds the threshold, the contig is assigned to the taxon that maximizes pass two score (“volume”) above the viral superkingdom. Pass one scores are only used as a barrier to entry for the LCA engine

### Code availability

The VirMAP software is open-source and available online at https://github.com/cmmr/VirMAP. VirMAP is implemented in Perl. For questions, comments, and notification of software updates, users can follow the VirMAP GitHub page. A document titled VirMAP_program_usage_and_options_table.xlsx describing command line options and a description of the software components used is available through GitHub.

### Data availability

The raw sequence datasets generated and analyzed during the current study are available through the Sequence Read Archive as follows: Viral Mock Community, BioProject ID PRJNA431646; NetoVir mock community, BioProject: PRJNA319556; Influenza dataset, BioProject ID PRJEB7888; Brazil dataset, BioProject ID PRJNA395784. The authors declare that all other data supporting the findings of this study are available within the article and its supplementary information files, or are available from the authors upon request.

## Electronic supplementary material

Supplementary Information

Description of Additional Supplementary Files

Supplementary Data 1

Supplementary Data 2

Supplementary Data 3

Supplementary Data 4

Supplementary Data 5

Supplementary Data 6

Supplementary Data 7

Supplementary Data 8

Supplementary Data 9

Supplementary Data 10

Supplementary Data 11

Supplementary Data 12

Supplementary Data 13

Supplementary Data 14

## References

[1] Lin HH, Liao YC (2017). drVM: a new tool for efficient genome assembly of known eukaryotic viruses from metagenomes. Gigascience.

[2] Lin J (2017). Vipie: web pipeline for parallel characterization of viral populations from multiple NGS samples. BMC Genom..

[3] Rampelli S (2016). ViromeScan: a new tool for metagenomic viral community profiling. BMC Genom..

[4] Segata N (2012). Metagenomic microbial community profiling using unique clade-specific marker genes. Nat. Methods.

[5] Tithi SS, Aylward FO, Jensen RV, Zhang L (2018). FastViromeExplorer: a pipeline for virus and phage identification and abundance profiling in metagenomics data. PeerJ.

[6] Truong DT (2015). MetaPhlAn2 for enhanced metagenomic taxonomic profiling. Nat. Methods.

[7] Yamashita A, Sekizuka T, Kuroda M (2016). VirusTAP: viral genome-targeted assembly pipeline. Front. Microbiol..

[8] Zhao G (2017). VirusSeeker, a computational pipeline for virus discovery and virome composition analysis. Virology.

[9] Menzel P, Ng KL, Krogh A (2016). Fast and sensitive taxonomic classification for metagenomics with Kaiju. Nat. Commun..

[10] Wood DE, Salzberg SL (2014). Kraken: ultrafast metagenomic sequence classification using exact alignments. Genome Biol..

[11] Modha, S. *MetaViC: Virus Metagenomics Pipeline for Unknown Host or in Absence of a Host Genome* https://github.com/sejmodha/MetaViC (2016).

[12] Wommack KE (2012). VIROME: a standard operating procedure for analysis of viral metagenome sequences. Stand. Genom. Sci..

[13] Li Y (2016). VIP: an integrated pipeline for metagenomics of virus identification and discovery. Sci. Rep..

[14] Roux S, Tournayre J, Mahul A, Debroas D, Enault F (2014). Metavir 2: new tools for viral metagenome comparison and assembled virome analysis. BMC Bioinforma..

[15] Conceicao-Neto N (2015). Modular approach to customise sample preparation procedures for viral metagenomics: a reproducible protocol for virome analysis. Sci. Rep..

[16] Padmanabhan, C., Zheng, Y., Li, R., Fei, Z. & Ling, K. S. Complete genome sequence of southern tomato virus naturally infecting tomatoes in Bangladesh. *Genome Announc.* **3**, 10.1128/genomeA.01522-15 (2015). 10.1128/genomeA.01522-15 PMC4698391 26722014

[17] Fischer N (2015). Evaluation of unbiased next-generation sequencing of RNA (RNA-seq) as a diagnostic method in influenza virus-positive respiratory Samples. J. Clin. Microbiol..

[18] Titus Brown, C., Howe, A., Zhang, Q., Pyrkosz, A. B. & Brom, T. H. A reference-free algorithm for computational normalization of shotgun sequencing data. *arXiv* 1203.4802v2 (2012).

[19] Bushnell B (2016). BBMap Short Read Aligner.

[20] Buchfink B, Xie C, Huson DH (2015). Fast and sensitive protein alignment using DIAMOND. Nat. Methods.

[21] Li D, Liu CM, Luo R, Sadakane K, Lam TW (2015). MEGAHIT: an ultra-fast single-node solution for large and complex metagenomics assembly via succinct de Bruijn graph. Bioinformatics.

[22] Li D (2016). MEGAHIT v1.0: A fast and scalable metagenome assembler driven by advanced methodologies and community practices. Methods.

