# Supplementary material for: Maximal viral information recovery from sequence data using VirMAP
Source: Nat Commun. 2018 Aug 10;9:3205. doi: 10.1038/s41467-018-05658-8 (PMC6086868; doi:10.1038/s41467-018-05658-8)
Supplement: Supplementary file 1 — Supplementary Information [file 41467_2018_5658_MOESM1_ESM.pdf]

Supplementary Table 1

Pipeline	Method	Description	Interface	Speed	Compute resource requirement	Ease of use	Scalability	Output versatility	Returns viral contigs
drVM	contig classifier	Read binning via mapping to genus-level viral databases followed by individual assembly of each genus bucket.	command line only	Fast	Medium	Medium	Low	Medium	Yes
FastViromeExplorer	Read classifier	Kallisto-based alignment to a viral database with a false-positive filter centered around skewed expected coverage.	command line only	Fast	Low	Medium	High	Medium	No
VirusSeeker	Read classifier	Heavy read-filtering prior to alignment to a viral database. Aligned reads are filtered by remapping to nr/nt.	command line only	Slow	High	Difficult	NA	Medium	No
Kaiju	Read classifier	Translated alignment against a BWT/FM-index database with LCAs calculated per-read.	command line and web based	Extremely fast	High	Medium	High	Medium	No
ViromeScan	Read classifier	Nucleotide alignment to a viral database and abundance calculated by taxonomic rank.	command line only	Fast	Low	Easy	High	Medium	No
VirusTap	Contig classifier	Extensive non-viral read filtering followed by an assembly and contig mapping to nr/nt.	web based only	Intermediate	NA	Easy	NA	Low	Yes
VIPIE	Contig classifier	Filtering of non-viral reads followed by an assembly. Viral references selected based on contigs mapped followed read remapping to viral references.	web based only	Fast	NA	Easy	Low	Medium	No
MetaPhlAn2	Marker gene classifier	Marker gene classification using an custom database.	command line and web based	Fast	Low	Easy	High	Medium	No
Standard Method	Contig classifier	Assembly followed by BLASTn and BLASTx to NCBI.	NA	Slow	Extremely high	Difficult	Low	High	Yes
VirMAP	pseudo-contig classifier	tiered mapping assembly from aggregate nucleotide and translated read alignments merged with a denovo assembly. BLASTn and BLASTx to a custom Genbank database.	command line only	Intermediate	High	Medium	High	High	Yes

Supplementary Table 1: Qualitative comparison of the bioinformatic pipelines used to process the Viral Mock Community dataset. The lack of informationally equivalent outputs prevented us from performing a quantitative analysis. Instead, we present a qualitative analysis highlighting what we consider are features relevant to the user. We evaluated interface (web based or command line accessibility), speed (time to process datasets), computer resource requirement (demand of local computational infrastructure) , ease of use (degree to which we could install and use the pipeline), scalability (ability to process a high volume of samples in a timely fashion), output versatility (ability to use outputs for a variety of downstream analyses), and the availability of viral contigs.

Supplementary Note 1

The pipelines presented in Table 1 were used to process the Influenza (PRJEB7888)[1] and Brazil (PRJNA395784) datasets with the exception of VirusTAP due to their server disallowing SRA uploads. All pipelines tested were run using default parameters with the exception of VirusSeeker which required modifications to the wrapper script allowing operation in a single machine. The default wrapper distributes jobs across an SGE cluster.

Observations on pipeline outputs:

drVM[2]
Influenza dataset:
Found influenza virus in every sample expected to have Influenza virus. Averaged 284 influenza calls per sample. Outputs provide enough information to subtype to H1N1 and H3N2. Missed Influenza C virus, and found Influenza B in the right sample.

Brazil dataset:
Averaged 34 viral genera calls per sample with an average of 1,163 individual references per sample.

FastViromeExplorer[3]
Influenza dataset:
Missed 5 samples with expected Influenza virus. Averaged 8 Influenza virus calls per sample. Outputs provide enough information to subtype to H1N1 and H3N2. Missed Influenza B and C virus.

Brazil dataset:
Averaged 58 viral genera calls per sample with an average of 66 individual references per sample.

VirusSeeker[4]
Influenza dataset:
Only 8 out of 25 datasets finished in the time allotted (48 hours). Influenza virus was found in all 8 samples. Outputs provide enough information to subtype to H1N1 and H3N2. Found Influenza B virus. Influenza C status unknown.

Brazil dataset:
No samples finished processing in the time allotted (48 hours).

Kaiju[5]
Influenza dataset:
Standard report does not expand viruses beyond super-kingdom (taxa id: 10239). All results are tabulated from Krona outputs. Found Influenza virus in all samples expected, as well as in five samples where it was unexpected. Averaged 162 Influenza calls per sample. Outputs do not consistently provide enough information to definitively subtype to H1N1 or H3N2. Able to find Influenza B and C virus.

Brazil dataset:
Averaged 2,272 individual viral calls per sample. Taxonomic splits per rank not possible due to mixed ranks per column in Krona outputs.

ViromeScan[6]
Influenza dataset:
Found Influenza virus in 7 out of 20 samples. Able to found Influenza B and C virus. Averaged 6 Influenza virus calls per sample. Outputs do not provide enough information to subtype to H1N1 and H3N2 due to taxonomic abstraction to only the species rank.

Brazil dataset:
Averaged 517 individual viral calls per sample. Averaged 466 species per sample.

VirusTap[7]
Influenza dataset:
Not attempted. SRA data are unsupported.

Brazil dataset:
Not attempted. SRA data are unsupported.

VIPIE[8]
Influenza dataset:
Failed 3 samples due to large input volume. Found Influenza virus in 11 out of 18 samples. Averaged 8 Influenza virus calls per sample. Outputs do not consistently provide enough information to subtype to H1N1 and H3N2. Missed Influenza C but Influenza B virus.

Brazil dataset:
Not attempted due to large input volume.

MetaPhlAn2[9]
Influenza dataset:
Found Influenza Virus in 20 out of the 20 samples. Outputs do not provide enough information to subtype to H1N1 and H3N2. It was able to find Influenza B and C virus.

Brazil dataset:
Averaged 46 viral species calls per sample.

Supplementary Note 2

Comparison of computational resources used during analysis

VMC Mock Community Dataset

VirMAP used 8.72 CPU hours to complete the full mock community set, while the standard approach used 6.02 CPU hours total. For the subsampled sets, VirMAP used 2.45, 1.85, and 1.40 CPU hours while the standard approach used 0.68, 0.27, and 0.08 CPU hours for the 10%, 1% and 0.1% subsampling trials, respectively. Due to the wide disparity in per base read coverage of the underlying genomes, the assembly step of the standard approach likely did not have enough read contiguity to assemble larger contigs. This is corroborated by very low percentage of viral bases constructed at the most extreme subsampling level.

NetoVir Mock Community Dataset

To analyze the full dataset, VirMAP used 24.99 CPU hours while the standard approach used 268.00 CPU hours. For the subsampled runs, VirMAP used an average of 8.21, 3.30, and 2.00 CPU hours per trial while the standard approach used an average of 125.73, 5.60, and 0.70 CPU hours per trial on average for the 10%, 1% and 0.1% subsampling trials, respectively. The large drop off in CPU time usage between the 10% and 1% trials is likely caused by coverage levels dropping below the minimum thresholds for assembly, resulting in fewer contigs to process. Additionally, with well characterized genomes, contigs are more likely to align to their parent genome with an e-value less than 1e-10, in which case the translated search is not performed in the standard approach used here.

Influenza Dataset

For the full influenza dataset, VirMAP used an average of 9.71 ± 4.32 CPU hours per sample, while the standard approach used an average of 112.42 ± 123.72 CPU hours per sample. The wide disparity in CPU hours used for the standard approach nearly perfectly correlated with the total assembly size (R^2 = .973), suggesting that alignments of contigs originating from non-viral contaminants consumed a large amount of CPU time. This highlights the advantage of VirMAP's internal filtering steps as those are performed with very quick high-identity alignments, quickly removing well known non-viral contigs and preventing them from entering the more exhaustive iterative improvement and high-sensitivity alignment phases.
For the subsampling trials, VirMAP used an average of 4.09 ± 1.70, 2.13 ± 1.27, and 1.18 ± 0.64 CPU hours per trial while the standard approach used 7.57 ± 9.09, 0.87 ± 0.88, and 0.26 ± 0.28 CPU hours for the 10%, 1% and 0.1% subsampling trials, respectively. A similar pattern can be seen in the standard approach's time usage for the subsampled set; the wide disparity and inability to find influenza virus at low coverage suggests that the only remaining contigs in a majority of the 1% and 0.1% trials are contaminants only. While the standard approach uses less CPU time in low coverage situations, this is only due to its inability to generate contigs to analyze.

Brazil sewage Dataset

For the full Brazil sewage dataset, the standard approach averaged 2937.42 ± 767.62 CPU hours per sample, while VirMAP used 69.03 ± 6.52 CPU hours. The extraordinary amount of CPU time used for the standard approach shows the intractability of exhaustively aligning every

contig from a generic metagenomic assembly when one is interested in viruses only. VirMAP efficiently uses CPU time in a targeted approach to effectively retrieve relevant viral information. The subsampled datasets showed a similar story. For the 10% set, the standard approach used 71.96 ± 34.29 CPU hours while VirMAP used 10.61 ± 1.67 CPU hours. For the 1% and 0.1% sets, the standard approach used 8.68 ± 3.62 and 0.68 ± 0.21 CPU hours while VirMAP used 2.65 ± 0.40 and 1.47 ± 0.15 CPU hours, respectively

Supplementary Note 3

Discussion of Dickeya virus Limestone misclassification

Due to the extreme level of read subsampling (0.1%), VirMAP was only able to recover subsections of the Dickeya virus Limestone genome. These fragmented genomes scored within 5% of three different database entries. One entry is Dickeya phage RC-2014, and the other is an incorrectly annotated database entry (Dickeya phage phiDP23.1) which shares 99% sequence identity across 97% of the genome with Dickeya virus Limestone. Since Dickeya phage phiDP23.1 is placed as a descendant of the unclassified phage taxon in NCBI's taxonomy tree, it affected VirMAP's taxonomic engine's decision making. Depending on which database entries were considered in the LCA, the reported taxonomy was underclassified to either "unknown phage" or "Myoviridae". These classification errors can be resolved by reducing the default 5% LCA fuzzing radius in the taxonomic classification engine. This threshold was empirically found to be an acceptable limit for all datasets tested by balancing over-classification and under-classification of contigs. However, the parameter is tunable by the user. Additionally, certain errors would be resolved with properly annotated database entries.

Supplementary Note 4

<u>Databases:</u>

Comprehensive Viral Database: Genbank Divisions used

47	gbvrl
3	gbphage

Comprehensive General Purpose Database: Genbank Divisions used

330	gbbct
356	gbcon
95	gbenv
15	gbhtc
152	gbinv
39	gbmam
283	gbpat
3	gbphg PHAGE – Separate processing also
143	gbpln
55	gbpri
30	gbrod
20	gbsts
9	gbsyn
1	gbuna
47	gbvrl VIRUS – Separate processing also
64	gbvrt

Genbank Divisons not used:

154	gbhtg
229	gbtsa
481	gbest
303	gbgss

Supplementary Note 5

Equations

1. Clustering

$$\frac{Readi}{Readc} < CR - (loge\left(\frac{Readu}{Readc}\right) * e/100$$

CR_i: Initial Cluster Radius (0.97 default)
$Read_i$: Shared reads (intersection) between $Read_c$ and $Read_u$
$Read_u$: Utilized reads
$Read_c$: Unique reads aligned to genomic entry
e: Euler's number, 2.718

$Read_i/Read_c < CR_i$ - ($\log_e$ ($Read_u/Read_c$) * e/100)

2. Taxonomic Classification

$$\frac{\frac{2}{3} - 0.5}{ln(dbRep)} + 0.5$$

dbRep = sum pass two fractional accuracy scores.

Each database entry produces a fractional score per taxon. This score is weighed by the database entry's per position bit-score against the best achieved per position bit-score across all taxa. Proteins are weighed tetrationally per base and nucleotides are weighed exponentially per base.

Fractional protein *dbRep* weight per base:

((Query bit-score/Query aligned length)/(best query bit-score/best scoring aligned length))^(3^3).

Fractional nucleotide *dbRep* weight per base:

((Query bit-score/Query aligned length)/(best query bit-score/best scoring aligned length))^3.

Supplementary Methods

Viral mock community

We generated a viral mock community (VMC) by combining seven different RNA and NA viruses (human adenovirus B, human adenovirus C, murine gammaherpesvirus, coxsackievirus B4 strain Tuscany, echovirus E13 strain Del Carmen, human poliovirus type 1 strain Mahoney, and rotavirus A) kindly provided by investigators of the Department of Molecular Virology and Microbiology, Baylor College of Medicine, in phosphate buffered saline solution. The VMC dataset is available at SRA (BioProject: PRJNA431646).

Data generation and processing

VMC was extracted using the MagMax Viral RNA Isolation Kit (Cat # AM1939, Thermo Fisher Scientific, Waltham, MA). Viral RNA was reverse transcribed using SuperScript II RT (Cat # 18064014, Thermo Fisher, Waltham, MA) and random hexamers. After short molecule and random hexamer removal with ChargeSwitch (Cat # CS12000, Thermo Fisher, Waltham, MA), molecules were amplified and tagged with a BC_{12}-V_8A_2 construct[10] using AccuPrime™ *Taq* polymerase and cleaned with ChargeSwitch kit. VMC amplicons were normalized, pooled, and made into an Illumina library without shearing. The library (150-600bp) was loaded in an Illumina HiSeq2500 (Illumina, Carlsbad, CA) and sequenced using the 2x150bp chemistry. Reads were demultiplexed into a sample bin using the barcode prefixing read-1 and read-2, allowing zero mismatches. Demultiplexed reads were further processed by trimming off barcodes, semi-random primer sequences, and Illumina adapters. This process utilized a custom demultiplexer and the BBDuk algorithm included in BBMap. The resulting trimmed dataset was compacted into a single file. VirMAP does not require the utilization of paired reads nor does it consider the pairing information at all. Once a working set of reads has been prepared, they are ready to be processed by VirMAP.

VirMAP consists of the following nine major steps (thresholds indicated are default and mostly tunable). A schematic of the pipeline is provided in Figure 1.

1) Read preparation: All input read files are numbered according to input order and processed into fasta format. Each read has its file input order number appended to the header, ensuring unique read names per read across all input files. Reads are then dereplicated with VSEARCH[11] using the derep_fulllength command with the sizeout option to include abundance information per read. After dereplication, reads are normalized using the khmer[12] software package's "normalize by median" function to a depth of 5 using a kmer of 31. Alternatively, bbnorm.sh from the BBMap suite of tools may be used. This produces a workable set of reads with nearly the same sequence information content as the dereplicated read set. Information loss can arise from filtering reads due to kmer coverage disparities. This manifests when the read actually contains globally unique kmers, but the median kmer abundance of the read is above the normalization cutoff. For example, this scenario could arise at a low abundance chimeric junction, containing information from two different genomic sources. Additionally, a read could be erroneously eliminated if >50% of the read's kmer set both exceed the normalization cutoff and can be explained through sources independent of the read's true origin. However, using a kmer size of 31 minimizes the likelihood of these events.

2) Read Alignment: Reads are aligned via nucleotide (BBMap)[13] and translated nucleotide (DIAMOND)[14] searches to a custom-built database consisting of all entries in Genbank's viral and phage divisions (gbvrl/gbphg) tagged with taxonomic information. The nucleotide to nucleotide alignments are restricted to >90% identity with secondary sites required to be >95% of the top-scoring hit. The translated nucleotide alignments are restricted to >80% identity in any one of the six possible frames of translation; all alignments within 8% identity of the top scoring alignment per read are reported. The initial >80% identity cutoff serves to recruit reads to

potentially homologous regions between similar proteins in related taxa if the database does not contain a good representative. The secondary 8% identity cutoff is set to preferentially use high identity alignments when clustering database hits, and to globally optimize the database entry used to represent the closest viral genomes present in the sample. This mitigates the case where the top scoring alignment information oscillates between available database entries. The secondary site threshold for BBMap serves the same purpose.

3) Clustering: Translated alignments are linked to their originating nucleotide entry via protein source annotation and contribute to each nucleotide entry's set of reads. If a protein entry has exclusively translated alignments, a marked nucleotide entry is created as a container for the protein alignments as clustering is done at the nucleotide accession level. Entries are sorted by the combined total unique read alignments in descending order. The top scoring nucleotide entry is automatically accepted and all of its corresponding aligned reads are considered utilized. Following this, lower abundance nucleotide entries are clustered via Jaccard distance between total utilized reads and the new genome's set of reads. The threshold of clustering is determined by an initial value of 0.97 followed by an adjustment. The adjustment is calculated by the log ratio of the total utilized reads and the current genome's set of associated reads, times *e*, divided by 100. If the Jaccard distance is below the adjusted threshold value, the genomic entry is accepted, as it provides a large number of unutilized reads. The threshold is calculated using Formula 1 (Supplementary Note 5).

As the number of utilized reads increases, the threshold of overlap for clustering decreases. This minimizes the acceptance of genomic entries that don't provide an appreciable amount of non-utilized reads. An eliminated entry's non-utilized reads are blacklisted and removed from further clustering consideration. The blacklist prevents acceptance of weak entries belonging to a similar taxonomy of an existing strong entry; as many blacklisted reads fail to align to the

strong entry’s parent genome due to sequencing or database errors. By transitively passing reads from protein alignments to the parent nucleotide, this clustering approach simultaneously evaluates nucleotide information for both coding and non-coding information and approximates a globally optimal minimal set of database entries for both types of alignments. The final output is a list of viral nucleotide database entries and their corresponding protein members.

4) Build pseudo-scaffolds: Read alignments to accepted database entries are used to build a consensus sequence. For each accepted entry, a pileup is independently built for the nucleotide entry and all associated protein entries in nucleotide space only. Because the originating read is known from the translated alignments, the translation process can be reversed, and a putative CDS per protein is constructed. The pileups are calculated per base for nucleotide entries and per codon for amino acid entries. The top scoring event per position is used. If there are multiple top-scoring hits, the positions covered by the event are masked with N’s. The final pileup result is a pseudo-scaffold. There are no restrictions on the length of the pseudo-scaffold, nor are there any requirements for the constituent reads to overlap. As a result, pseudo-scaffolds can be generated with less than 1X coverage. However, regions must have a clear consensus for >25% of the total positions covered by multiple reads. In essence, if there are multiple read alignments that overlap position-wise, the sequence content must somewhat agree. Positions with no read alignments are masked with an N instead of interpolating in the underlying reference’s sequence information. Insertions and deletions are not considered at this stage and are eliminated; thus, all positions are kept coherent to the originating reference positions. This is to minimize any required shuffling of coordinates in the upcoming super-scaffolding stage.

5) Build super-scaffold: Protein pseudo-scaffolds are super-scaffolded onto the parent nucleotide pseudo-scaffold according to the annotated positions of their coding sequence. Super-scaffolding allows VirMAP to capture viruses whose nucleotide sequences have diverged

significantly from the closest database entry while their amino acid sequences have not. If positions between the nucleotide and protein pseudo-scaffolds disagree, the nucleotide scaffold takes precedence. After construction, dereplicated reads are aligned to the final set of super scaffolds, and each one is reconstructed based on alignment pileups. Insertions and deletions are taken into account at this phase in order to produce more accurate super-scaffolds. These finalized super-scaffolds represent VirMAP's best effort at a mapping based pseudo-assembly to reconstruct known viral information.

6) De novo assembly: VirMAP utilizes two programs for de novo metagenome assembly, MEGAHIT[15,16] and BBMap's tadpole assembler[13]. By default, multiple tadpole assemblies across a range of kmer sizes are generated, with the option of using MEGAHIT, or both assemblers in a single run. However, threaded runs of MEGAHIT are non-deterministic. The associated VirMAP assembly options, including 'sensitive' modes, are detailed in (Supplementary Data 14). Resulting contigs are deduplicated using the dedupe.sh script from the BBMap suite to produce a minimal set of minimally overlapping contigs. All contigs under 500bp are not reported by any assembly methods used in VirMAP. Contigs generated during de novo assembly are merged with the pseudo-assembly from step 5. Any non-merged contigs are kept for further analysis and tagged with the assumed taxa of virus (NCBI taxonomy ID:10239), as they may represent unknown viruses. In downstream pipeline steps, sequences from the merged dataset will be simply be referred to as contigs, unless otherwise specified.

7) Filtering: Public viral databases are contaminated with non-viral sequences from a variety of sources including vector sequences, host sequences, or assembly errors incorporating extra-genomic information. To identify and remove contigs that are likely non-viral, two filters are used. Initially, all contigs with lower than 5 bits of entropy per triplet are removed. Remaining contigs are aligned to a nucleotide database constructed from 16 of Genbank's 20 divisions, via

megablast[17], to a depth of 1,000 entries. Contigs with >50% alignment score to a non-viral taxon are eliminated. Alignment scoring is calculated using the same strategy for taxonomic classification described in section 9 below. All remaining contigs are queried against a protein database constructed from 16 of Genbank's divisions, via DIAMOND's blastx function, to a depth of 1,000 entries. Each contig is evaluated per base for an originating taxon, and once again, contigs with >50% alignment score to a non-viral taxon are eliminated.

8) Iterative improvement (merge, gap-fill, and extend): Prior to main cycle of the iterative improvement process, contigs are subjected to an initializing merge with the algorithm described below, but using a word size of 51. Subsequently, contigs are clustered using kmer overlap where any two contigs are clustered if they are within 10% of each other in length, and share >99% kmer overlap. The initial step of the iterative improvement algorithm is merging, which is itself an iterative process. The merging cycle begins by reverse complementing and tagging the full contig set, and then appending that reverse complemented set to the original. Subsequent merge cycles begin the same way, but use the merged contig set yielded by the previous cycle. The resulting contig super set is self-aligned using a strand-specific ungapped megablast[17] with a word size of 31. Any contig that is wholly contained within another contig at >99% identity is merged into the larger contig. Remaining contig alignments are evaluated pairwise for mutual reciprocity since each aligned contig in the set should exist as both subject and query. An unbalanced alignment for a contig pair removes that pair from downstream merging considerations, and any given contig can only undergo a single merge event per cycle. In order for two contigs to merge, they must not exceed 3% column mismatch within the overlapping region, or exceed 4% column mismatch when considering frame-coherent alignments to an 'N'. A frame coherent alignment to an 'N' results in a +.5 when calculating column identities. This can be imagined as two contigs with N's opposite non-N's like a zipper, except for a slight overlap at the edges of each contig. Any N opposed to known sequence information "fits"

despite not being a true alignment. The half score is given to reflect that. The merging cycle is considered complete when no contigs merge during a pass. The merging cycle is performed for each iterative improvement pass.

After each merging cycle, the dereplicated read set (from step 1 above) is aligned to the set of merged contigs using BBMap, initially at 80%. A mapping assembly is carried out using solely read alignment information, discarding unaligned regions or contigs. In the event of reads aligning chimerically, only the portion of the read with the longest aligning segment is used, and the shorter chimeric end is discarded. The alignment identity threshold is raised by 3.4%, for the first 5 iterations, topping out at 97% identity for iterations 6+. The iterative improvement process is considered complete when all contigs converge, or 40 cycles have been attempted. Convergence is defined as 0% change between cycles 1-2, less than 0.5% change between cycles 3-5, or less than 1% change between cycles 6-40. Additionally, beyond cycle 2, if a contig re-builds as a previously seen version of itself, it is considered converged.

9*)* Taxonomic Classification: The remaining set of contigs are aligned to the databases described in step 7 via blastn (word size 17) and a two-step translated alignment using DIAMOND's blastx function. For the translated search, contigs are initially chunked into segments of 100bp overlapping by 50bp and all alignments below a 1e-5 are recorded. Any aligning segment of a contig is then masked, and the masked contig set is then aligned as a whole using a 0.1 e-value threshold. Up to 100,000 alignments per high-scoring segment pair are stored. For each contig, a maximum observed bit score per base for each subject taxon is calculated from the alignments. Only taxa with at least 1 base scoring within 10% of the highest observed score at that base's position are kept for downstream analysis, however, all scores of such taxa are considered for alignment scoring. Aggregate bit scores per taxa are calculated by

summing the total best case bits per base per taxa across all positions. This represents the best-case alignment score per taxonomic ID independent of subject contiguity.

A second pass over all contig alignments is performed. Any alignment whose taxa ID did not appear within 10% of the best bit score per base at any position are ignored. Each individual alignments' actual bit per base per taxa score is weighed against the best-case bit per base score across all taxa covering that position. The ratio of the actual bit score to best case bit score is transformed by raising it to the power of 3 for nucleotide entries and 27 for protein entries, thereby collapsing the score contribution of suboptimal alignments. This represents a database entry's fractional increase of information weighed by bit score ratio (referenced in determineTaxonomy.pl line 297). For example, in any given query range, a top scoring nucleotide alignment of 1.80 bits per base would contribute a score of "1" per base covered, however, an alignment of 1.70 bits per base covering the same query range would contribute a bit-ratio score of (1.70/1.80)^3 per aligning base. Due to the higher conservation rates observed for protein sequences, lower bit score ratios are penalized tetrationally as opposed to exponentially. For example, an aligning segment with a score of .60 bits per base scaled against a top scoring protein alignment of .70 bits per base would contribute a (.60/.70)^(3^3) bit-ratio score per aligning base. Additionally, nucleotide segments with no protein alignment covering the same segment are tetrationally weighted. To calculate an alignment score per contig, all bit-ratio score contributions per base per taxa across all alignments are summed, which approximates the volume of database information (dbRep) per taxa per contig, weighted by aggregate alignment quality, where suboptimal alignments are scaled as previously described. This helps the LCA engine (described below) resolve situations where a missannotated database entry is the best match, by weighing all alignments from a relevant taxa ID (top 10% taxa per base score) instead of relying only on top-hit. For the filtering mode, all unaligned bases are considered to be of viral origin and weighted with the top bit-ratio contributions per

base across all alignments. This ensures that a contig must have a non-viral taxon as the best scoring taxa over at least 50% of its length before it is considered for removal.

Contigs derived from de novo assemblies that contain at least 5.5bits of information per triplet and are unaligned to any sequence across at least 80% of the contig length are not filtered. These contigs are tagged as containing high unknown information content, and no further analysis is performed. Remaining contigs are subjected to a lowest common ancestor (LCA) analysis. For the LCA analysis, all taxa within a calculated radius of the top nucleotide and protein aggregate bit scores are analyzed. Each radius is dynamically calculated based on the total alignment information content and the relative accuracy of the alignments. The radius will expand slightly as alignment accuracy goes down and contract slightly as total aligning length goes up. All taxa that are within both radii are considered in the LCA engine. In the event that no such taxa satisfy both radii, each radius is considered separately and contributes their top scoring taxa to the LCA pool.

Any taxonomic nodes supplied to the LCA engine are considered equally valid taxonomic origins. To find the best LCA, each node contributes its total database volume information to its parent, and the first taxonomic node to reach a calculated majority threshold (Supplementary Note 5) and referenced in determineTaxonomy.pl line 1464) relative to the total database representation of the input nodes -- while traversing to root from the starting node -- is considered to be the taxonomic origin of the contig. This helps resolve the taxonomic ID decision in ambiguous cases where there are two similarly scoring taxa of nearly equivalent alignment information or where database coverage of the true origin is low. Maximum possible aggregate bit scores are calculated assuming perfect self-alignment. Contigs having aggregate bit scores <80% of the calculated maximum are tagged as divergent sequences. Contigs with aggregate bit scores <1000 bits are considered to have low information content and are tagged

as “weak”. If the top 14% of aggregate bit scores of a contig contain a taxon that is of non-viral origin, the contig is tagged accordingly. Additionally, contigs are tagged with all of their relevant attributes (e.g. aggregate bit score, number of aligned reads, total proportion of non-n letters, max viral and protein nucleotide and protein bit scores).

Interpreting taxonomic alignments

Final viral contigs are tagged with their determined taxonomic origin (taxId), best aggregate bit score (maxScore), best possible aggregate bit score (maxPossibleScore), best possible aggregate bit score of aligning sections (maxAlign), best possible aggregate alignment subscores for both protein and nucleotide alignments (maxProtAlign, maxNuclAlign), best actual aggregate alignment subscores for both protein and nucleotide alignments (protScore, nuclScore), percentage of contig bases not occupied by an “N” (letterOccupancy), sequence length (length), number of aligning reads (size), and any combination of the following tags: 1) “weak” [score <1000], 2) “highDivergence” [score <80% of theoretical maximum], 3) “High (non-viral superkingdom)” [A taxa outside of the viral superkingdom was considered in the LCA], 4) “highUnknownInformation” [>500bp, >5.5 bits of information per triplet, >80% unaligned across total length]. 5) “potentialMisannotations” [super majority not reached (majority threshold < (taxa dbRep / total dbRep) < 66%), majority threshold not reached (50% < (taxa dbRep / total dbRep) < majority threshold), majority not reached [(taxa dbRep / total dbRep) < 50%)].

Standard Assembly and Mapping

Reads were assembled with MEGAHIT (default parameters) followed by blastn of resulting contigs (default parameters and reporting). The reported taxa represent the top hits based on e-score, if multiple high-scoring segment pairs had the same e-score, the taxon with the highest bit score was reported. If the top bit scores were the same, but the reported taxa were different, a naive lowest common ancestor (LCA) was performed and reported. If there were no blastn

results of e-score less than 1e-10, the contig was subjected to blastx (default settings for blastx-fast), and a taxon was determined from the results the same way as described above for blastn.

Database

VirMAP employs comprehensive nucleotide and protein databases totaling approximately 116 gigabytes in its usable form. The databases used in VirMAP for initial mapping steps and construction of pseudo-scaffolds are composed of non-dereplicated nucleotide and amino acid sequences found in gbvrl and gbphg. Each entry is tagged with the taxonomic ID of its origin. Furthermore, amino acid sequences are tagged with their respective CDS coordinates. Viral databases are kept in a non-dereplicated state to preserve identical proteins across different taxa and database entries as well as their associated coordinates. The databases used in VirMAP to filter and determine the taxonomic origin of the contigs are composed of 16 GenBank divisions. Both nucleotide and amino acid databases are dereplicated, except for the gbvrl and gbphg divisions. Database updates are synchronized with GenBank releases (quarterly) and previous versions are kept for up to one year.

Computing requirements

While not extraordinarily resource intensive, VirMAP does contain certain steps that are RAM intensive. The initial mapping to the viral nucleotide database consumes on average 25GB of RAM. The normalization step can require large amounts of RAM if the input set of reads is very deep and very wide. However, most runs of VirMAP should fit on a single machine with 32GB of RAM with a CPU capable of processing 4 simultaneous threads.

The time needed to run VirMAP is highly dependent on the quantity and sequence similarity of the reads to database entries, and on the number of closely related database entries.

Additionally, the iterative improvement phase can consume a lot of time, especially if there are low coverage viruses that are very divergent from known database sequences.

Supplementary References

1. Fischer, N. *et al.* Evaluation of Unbiased Next-Generation Sequencing of RNA (RNA-seq) as a Diagnostic Method in Influenza Virus-Positive Respiratory Samples. *J Clin Microbiol* **53**, 2238-2250, doi:10.1128/JCM.02495-14 (2015).
2. Lin, H. H. & Liao, Y. C. drVM: a new tool for efficient genome assembly of known eukaryotic viruses from metagenomes. *Gigascience* **6**, 1-10, doi:10.1093/gigascience/gix003 (2017).
3. Tithi, S. S., Aylward, F. O., Jensen, R. V. & Zhang, L. FastViromeExplorer: a pipeline for virus and phage identification and abundance profiling in metagenomics data. *PeerJ* **6**, e4227, doi:10.7717/peerj.4227 (2018).
4. Zhao, G. *et al.* VirusSeeker, a computational pipeline for virus discovery and virome composition analysis. *Virology* **503**, 21-30, doi:10.1016/j.virol.2017.01.005 (2017).
5. Menzel, P., Ng, K. L. & Krogh, A. Fast and sensitive taxonomic classification for metagenomics with Kaiju. *Nat Commun* **7**, 11257, doi:10.1038/ncomms11257 (2016).
6. Rampelli, S. *et al.* ViromeScan: a new tool for metagenomic viral community profiling. *BMC Genomics* **17**, 165, doi:10.1186/s12864-016-2446-3 (2016).
7. Yamashita, A., Sekizuka, T. & Kuroda, M. VirusTAP: Viral Genome-Targeted Assembly Pipeline. *Front Microbiol* **7**, 32, doi:10.3389/fmicb.2016.00032 (2016).
8. Lin, J. *et al.* Vipie: web pipeline for parallel characterization of viral populations from multiple NGS samples. *BMC Genomics* **18**, 378, doi:10.1186/s12864-017-3721-7 (2017).
9. Truong, D. T. *et al.* MetaPhlAn2 for enhanced metagenomic taxonomic profiling. *Nat Methods* **12**, 902-903, doi:10.1038/nmeth.3589 (2015).
10. Clem, A. L., Sims, J., Telang, S., Eaton, J. W. & Chesney, J. Virus detection and identification using random multiplex (RT)-PCR with 3'-locked random primers. *Virol J* **4**, 65, doi:10.1186/1743-422X-4-65 (2007).
11. Rognes, T., Flouri, T., Nichols, B., Quince, C. & Mahe, F. VSEARCH: a versatile open source tool for metagenomics. *PeerJ* **4**, e2584, doi:10.7717/peerj.2584 (2016).
12. Crusoe, M. R. *et al.* The khmer software package: enabling efficient nucleotide sequence analysis. *F1000Res* **4**, 900, doi:10.12688/f1000research.6924.1 (2015).
13. Bushnell, B. BBMap short read aligner. *University of California, Berkeley, California. URL http://sourceforge. net/projects/bbmap* (2016).
14. Buchfink, B., Xie, C. & Huson, D. H. Fast and sensitive protein alignment using DIAMOND. *Nat Methods* **12**, 59-60, doi:10.1038/nmeth.3176 (2015).
15. Li, D., Liu, C. M., Luo, R., Sadakane, K. & Lam, T. W. MEGAHIT: an ultra-fast single-node solution for large and complex metagenomics assembly via succinct de Bruijn graph. *Bioinformatics* **31**, 1674-1676, doi:10.1093/bioinformatics/btv033 (2015).
16. Li, D. *et al.* MEGAHIT v1.0: A fast and scalable metagenome assembler driven by advanced methodologies and community practices. *Methods* **102**, 3-11, doi:10.1016/j.ymeth.2016.02.020 (2016).
17. Altschul, S. F., Gish, W., Miller, W., Myers, E. W. & Lipman, D. J. Basic local alignment search tool. *J Mol Biol* **215**, 403-410, doi:10.1016/S0022-2836(05)80360-2 (1990).
