# Supplementary material for: Maximal viral information recovery from sequence data using VirMAP
Source: Nat Commun. 2018 Aug 10;9:3205. doi: 10.1038/s41467-018-05658-8 (PMC6086868; doi:10.1038/s41467-018-05658-8)
Supplement: Supplementary file 2 — Description of Additional Supplementary Files [file 41467_2018_5658_MOESM2_ESM.pdf]

Description of Additional Supplementary Files

File Name: **Supplementary Data 1**
Description: **Analysis of the Viral Mock Community, the NetoVir mock community, the Influenza, and the Brazil datasets with VirMAP and the Standard Approach.** Comparison of the Standard Approach and VirMAP's output, runtime, and computational results.

File Name: **Supplementary Data 2**
Description: **VirMAP analysis of the Viral Mock Community.** Analysis of the Viral Mock Community including subsampled fractions with VirMAP.

File Name: **Supplementary Data 3**
Description: **Standard assembly and mapping analysis on the Viral Mock Community.** Analysis of the Viral Mock Community including subsampled fractions with the Standard Approach.

File Name: **Supplementary Data 4**
Description: **VirMAP analysis of the NetoVir mock community.** Analysis of the NetoVir mock community including subsampled fractions with VirMAP.

File Name: **Supplementary Data 5**
Description: **Standard assembly and mapping analysis of the NetoVIR mock community.** Analysis of the NetoVir including subsampled fractions with the Standard Approach.

File Name: **Supplementary Data 6**
Description: **VirMAP analysis of a published dataset containing 20 influenza positive samples.** VirMAP analysis of a dataset submitted by University Medical Center Hamburg-Eppendorf on 2015-05-22 under the title Unbiased metagenomic RNA sequencing (UMERS) of 20 respiratory specimens (BAL, sputum and swab) of patients with seasonal influenza infection and five BAL samples from patients with influenza-negative pneumonia, (BioProject PRJEB7888).

File Name: **Supplementary Data 7**
Description: **Detailed description of the results obtained after analyzing 20 influenza positive samples with VirMAP.** VirMAP analysis of a dataset submitted by University Medical Center Hamburg-Eppendorf on 2015-05-22 under the title Unbiased metagenomic RNA sequencing (UMERS) of 20 respiratory specimens (BAL, sputum and swab) of patients with seasonal influenza infection and five BAL samples from patients with influenza-negative pneumonia, (BioProject PRJEB7888), including a detailed comparison of the authors' results and those obtained by VirMAP.

File Name: **Supplementary Data 8**
Description: **Comparison of the results obtained after analyzing 20 influenza positive samples with VirMAP and the Standard Approach.** Comparison of the results obtained after analyzing 20 influenza positive samples with VirMAP and the Standard Approach including subsampled fractions.

File Name: **Supplementary Data 9**
Description: **Standard assembly and mapping analysis of 20 influenza positive samples.** Standard assembly and mapping analysis of Influenza dataset including subsampled fractions.

File Name: **Supplementary Data 10**
Description: **Analysis of 4 sewage samples with VirMAP.** VirMAP analysis of a complex metagenomic dataset submitted by the University of Brasilia on 2017-08-01 under the project title "Viral diversity of the Federal District of Brazil" (BioProject: PRJNA395784), including subsampled fractions.

File Name: **Supplementary Data 11**
Description: **Analysis of 4 sewage samples with the Standard Approach.** Analysis of a complex metagenomic dataset submitted by the University of Brasilia on 2017-08-01 under the project title "Viral diversity of the Federal District of Brazil" (BioProject: PRJNA395784), including subsampled fractions with the Standard Approach.

File Name: **Supplementary Data 12**
Description: **Analysis of 4 sewage samples with the Standard Approach after applying an alignment filter.** Analysis of a complex metagenomic dataset submitted by the University of Brasilia on 2017-08-01 under the project title "Viral diversity of the Federal District of Brazil" (BioProject: PRJNA395784), including subsampled fractions with the Standard Approach after applying a 300-bit alignment threshold

File Name: **Supplementary Data 13**
Description: **Comparison of the results obtained after analyzing 4 sewage samples with VirMAP and the Standard Approach.** Raw and Filtered results after analyzing 4 sewage samples with VirMAP and the Standard Approach including subsampled fractions.

File Name: **Supplementary Data 14**
Description: **Required software dependencies and a brief description of commands for VirMAP.** Summary of software dependencies, the activities related to each of them, and the options allowed by VirMAP with brief descriptions.
